# A long-term monitoring dataset of fish assemblages in rocky tidepools on the northern coast of Taiwan

**DOI:** 10.1038/s41597-020-0425-7

**Published:** 2020-03-09

**Authors:** Lin-Tai Ho, Shen-Chih Wang, Kwang-Tsao Shao, I-Shiung Chen, Hungyen Chen

**Affiliations:** 1 National Museum of Marine Science and Technology, Keelung, 20248 Taiwan; 2 Institute of Marine Biology, National Taiwan Ocean University, Keelung, 20224 Taiwan; 3 Biodiversity Research Center, Academia Sinica, Taipei, 11529 Taiwan; 4 Center of Excellence for the Oceans, National Taiwan Ocean University, Keelung, 20224 Taiwan; 5 Department of Agronomy, National Taiwan University, Taipei, 10617 Taiwan

**Keywords:** Biodiversity, Conservation biology, Ichthyology, Marine biology

## Abstract

The assemblage of fish species in the rocky intertidal zone is highly affected by the destructive impact of human activities and has an extended impact on land-sea interactions. There are a few long-term research projects that have focused on rocky intertidal ecosystems, especially on the resident fish community. Here, we describe a long-term time series dataset of fish collected by counting the number of anesthetized fishes at sampling stations in rocky tidepools in the intertidal zones on the northern coast of Taiwan. The species assemblages were monitored seasonally at three stations from 1999 to 2018. In total, 144 samples containing 1,577 individuals belonging to 106 species were recorded in the surveys. The resulting data can be used as background information for conservation and resilience studies of the fish community in coastal areas and to establish reasonable conservation strategies. This study presents valuable data to ecologists and fisheries biologists interested in understanding the temporal patterns of species abundance, richness, and composition in relation to environmental factors, climate change, and anthropogenic pressures.

## Abstract (summary)

**Table:** 

Measurement(s)	Fish
Technology Type(s)	Manual Count
Factor Type(s)	time of data collection on species assemblages
Sample Characteristic - Environment	tidal pool • coast
Sample Characteristic - Location	Taiwan Island

Machine-accessible metadata file describing the reported data: 10.6084/m9.figshare.11898174

## Background & Summary

The rapid disappearance of species and dwindling of biodiversity on Earth is an important issue that will affect the sustainable existence of human beings and is a great challenge in this century. To explore the underlying mechanisms of community change and evaluate the effectiveness of conservation measures, long-term datasets of species abundance and diversity are particularly important.

To date, Taiwan’s investment in long-term ecological monitoring of fish communities has proven inadequate. The surveys and collected data are usually short-term, fragmented, incomplete, and improperly archived, resulting in a dearth of baseline ecological data. Perhaps the best example of a long-term marine ecological dataset is the survey of fish assemblages in the seas around the nuclear power plants of northern Taiwan, which contains over 30 years of monthly or seasonally gathered data. These data may reflect the community changes of surface and benthic fish in the local coastal subtropical zone^1–5^. Chen *et al*. described the long-term time series datasets of fish assemblages at nuclear power plants in northern Taiwan collected by impinged fish sampling at cooling water intake screens^2^, and collected by trammel net fish sampling and observed by an underwater diving visual census near thermal discharges^3^. Using these data, the same research team proposed an index of phylogenetic skew to describe the temporal variation of species composition of the assemblages over 25 years^4^, and analyzed the stochastic seasonality for the most abundant species, *Diodon holocanthus*, in northern Taiwan using monthly data over 11 years^5^. In Taiwan, the monitoring of projects concerning the ecological restoration of oil pollution, sewage pollution, cold damage, or cold-water intrusion usually only supplies short-term data. The investigation into the effects of sewage treatment near a power plant in Taipei using fixed fishing gill nets was conducted during a 12-year period^6^. In addition, the above mentioned studies were conducted in the sub-tidal zone, and there is no current long-term study of the fish assemblages in the intertidal zone in Taiwan. The intertidal zone suffers the highest impact of destructive anthropogenic influence and is the primary area of land-sea interaction.

Studies of fish communities in the intertidal zone have primarily been conducted in the reef coasts of the Northeast Pacific (particularly in North America) and western Europe, and there are few reports from tropical and subtropical areas^7,8^. Tidepools represent a unique ecosystem of rocky intertidal zones that provide a study site to investigate the variation in fish populations and community^9^. Many of these studies have investigated the temporal and spatial dynamics of community structures^10–14^, primarily in the context of the stability and resilience of communities^7,15^. Although reports of seasonal and annual changes in community structure have been published, relatively few of these studies are long-term surveys^10–12,16–18^.

In this study, we describe a long-term time series dataset of fish collected by counting the number of anesthetized fishes at sampling stations in rocky tidepools in the intertidal zone on the northern coast of Taiwan. Time series data provide a valuable resource for elucidating the long-term trends in fish community ecology and the intertidal ecosystem of this area. Additionally, these data provide the background information for the conservation and resilience of the fish community in coastal areas. These data can also be used by ecologists and fisheries biologists interested in understanding the temporal patterns of species abundance and composition in relation to environmental factors, climate change, and anthropogenic pressures.

## Methods

**Fig. 1:**
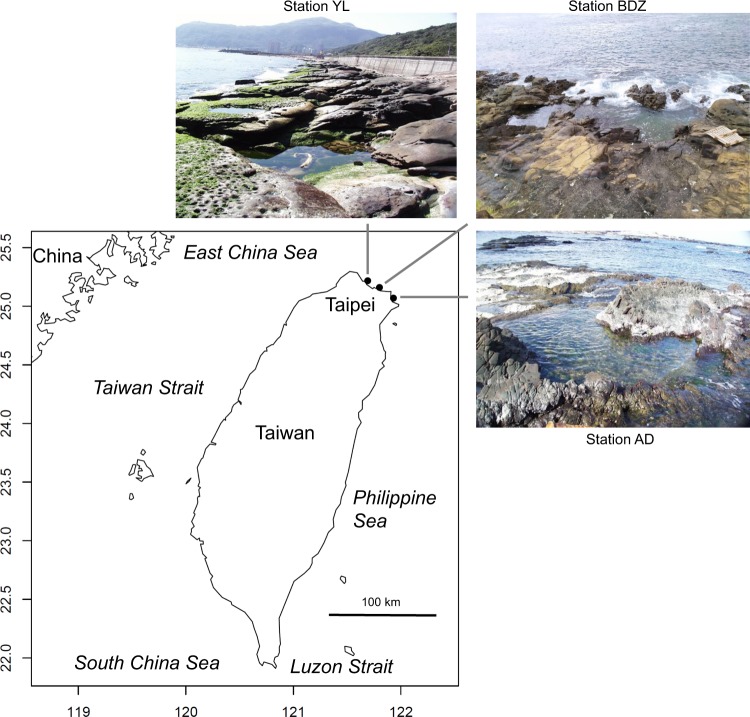
Map and photos of sampling stations (Stations YL, BDZ, and AD).

The fish community composition data were collected at three sampling stations in rocky tidepools in the intertidal zone on the northern coast of Taiwan (Fig. 1). Station YL (WGS84: 25°11′60″N, 121°43′31″E) was located near Yehliu in New Taipei City, station BDZ (25°08′35″N, 121°48′13″E) was located near Badouzi in Keelung City, and station AD (25°03′29″N, 121°55′38″E) was located near Aodi in New Taipei City. Station BDZ was located about 12 km eastward of Station YL and about 13 km westward of station AD.

Fish samples were collected during four seasons (January to March, April to June, July to September, and October to December) at Station YL (from October 1999 to November 2018), Station BDZ (from January 1999 to October 2018), and Station AD (from October 1999 to December 2018). Data were collected by counting the number of anesthetized fishes, and the operations were carried out during low tide of the day, with an average operation time of about 1 h. The sampling days were chosen with reference to meteorological conditions obtained from Central Weather Bureau. The anesthetic used in the intertidal fish collection was clove oil mixed with alcohol in a ratio of 1:2 (clove oil:alcohol) and diluted to a concentration of 100 ppm. The anesthetic was poured into the tidepool and well mixed. After the anesthetic took effect, three laboratory members used fishing nets to catch fishes that were swimming slowly. Fifteen minutes later, fishes that remained hidden in the cracks of the rock or at the bottom of the pool were collected by hand carefully. The tidepools were all completely flushed at high tide, so fishes were recruited into the pools in two to three days. Effects of removing all fish from the tidepool have not been observed during the studying period. The collected fish were placed in sealed packs and transported back to the laboratory on ice. The identification and counting of collected fishes were conducted in the laboratory. Fishes were identified personally by fish taxonomist Mr. Lin-Tai Ho, by referring to information in Fishbase (http://fishbase.org), Taiwan Fish Database (http://fishdb.sinica.edu.tw), and identification keys^19^. The identification was considered reliable because the number of recorded species was low and primarily consisted of common species. Sampling and species identification methods were consistent across the years of the study.

## Data Records

The dataset includes 144 samples collected at tidepools and 47, 50, and 47 samples from station YL, station BDZ, and station AD, respectively. The data are represented as a list of species names and their abundance collected at the three stations^20^. The data contain 106 rows (species) and 144 columns (station, year, and month). The fish assemblage data are provided in a CSV file.

## Technical Validation

**Fig. 2:**
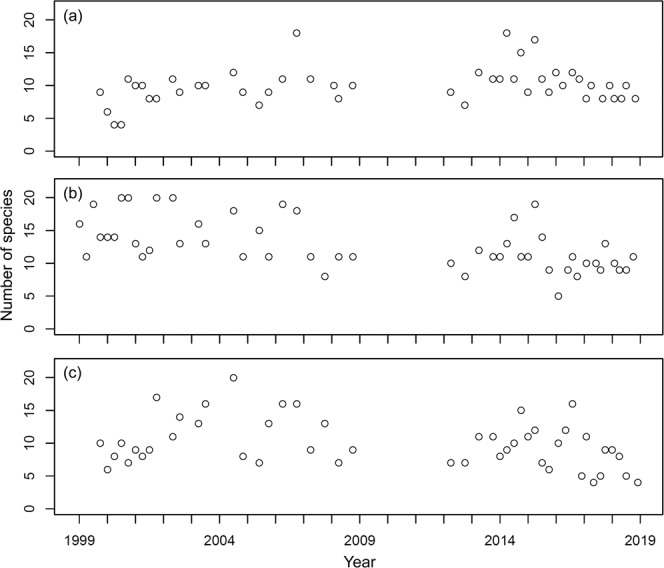
Temporal variations of number of species at sampling stations during the period 1999–2018. (**a**) Station YL, (**b**) Station BDZ, and (**c**) Station AD.

**Fig. 3:**
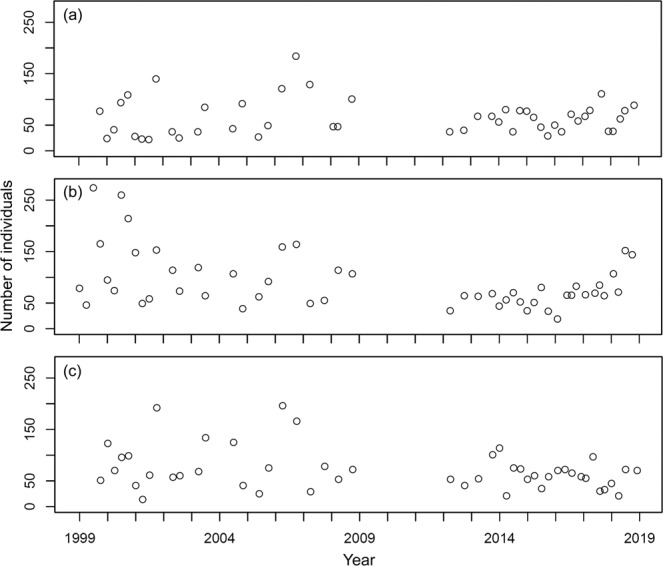
Temporal variations of number of individuals at sampling stations during the period 1999–2018. (**a**) Station YL, (**b**) Station BDZ, and (**c**) Station AD.

**Fig. 4:**
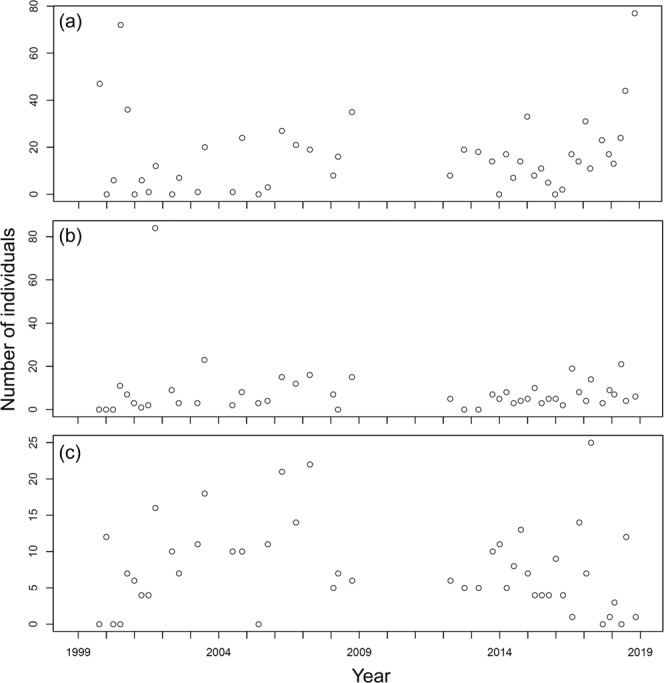
Temporal variations of number of individuals for abundant species at station YL during the period 1999–2018. (**a**) *Bathygobius fuscus*, (**b**) *Abudefduf vaigiensis*, and (**c**) *Istiblennius edentulus*.

**Fig. 5:**
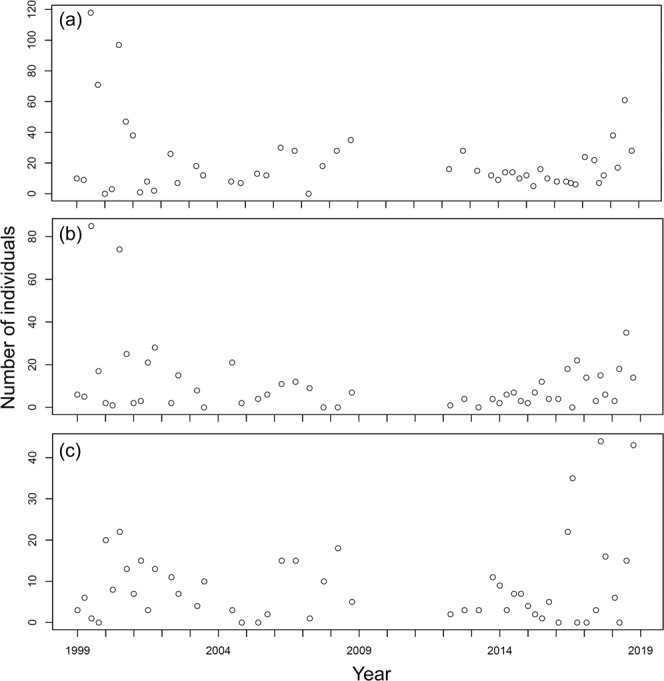
Temporal variations of number of individuals for abundant species at station BDZ during the period 1999–2018. (**a**) *Bathygobius fuscus*, (**b**) *Abudefduf vaigiensis*, and (**c**) *Istiblennius edentulus*.

**Fig. 6:**
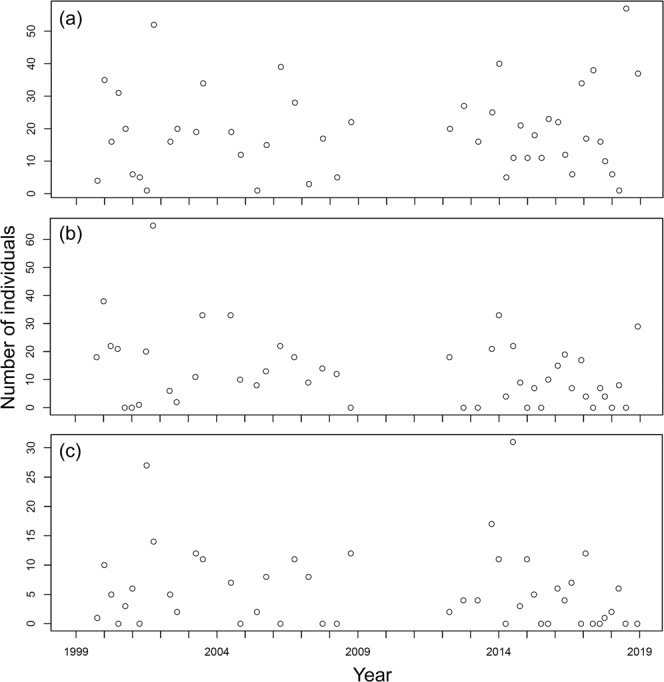
Temporal variations of number of individuals for abundant species at station AD during the period 1999–2018. (**a**) *Bathygobius fuscus*, (**b**) *Istiblennius dussumieri*, and (**c**) *Istiblennius lineatus*.

At station YL, 470 individuals belonging to 65 species were collected; the number of species collected per sample ranged between 4 and 18 and averaged 10.00 ± 2.84 (s.d.) in the samples (Fig. 2a), while the number of individuals collected per sample ranged between 22 and 184 and averaged 64.66 ± 34.80 (s.d.) (Fig. 3a). For station BDZ, 639 individuals belonging to 82 species were collected. The number of species collected per sample ranged between 5 and 20 and averaged 12.78 ± 3.79 (s.d.) in the samples (Fig. 2b), while the number of individuals collected per sample ranged between 19 and 274 and averaged 91.50 ± 55.21 (s.d.) in the samples (Fig. 3b). For station AD, 468 individuals belonging to 64 species were collected. The number of species collected per sample ranged between 4 and 20 and averaged 9.96 ± 3.71 (s.d.) (Fig. 2c), while the number of individuals collected per sample ranged between 14 and 196 and averaged 71.32 ± 40.80 (s.d.) (Fig. 3c). In total, 1,577 individuals belonging to 106 species were collected at the three stations. The number of species collected per sample overall ranged between 4 and 20 and averaged 10.95 ± 3.71 (s.d.) at the three stations, while the number of individuals collected per sample ranged between 14 and 274 and averaged 45.86 ± 76.15 (s.d.) at the three stations. The number of species collected at the three stations fluctuated seasonally from 1999 to 2018 (Fig. 2). The seasonal fluctuations of the number of individuals collected from 1999 to 2018 are shown in Fig. 3. The most abundant species in the samples are *Bathygobius fuscus*, *Abudefduf vaigiensis*, and *Istiblennius edentulus* at stations YL and BDZ, separately, and *Bathygobius fuscus*, *Istiblennius dussumieri*, and *Istiblennius lineatus* at station AD. The long-term temporal fluctuations of the number of individuals for these abundant species are shown in Figs. 4–6.
